# Risk factors and outcomes of inpatients with carbapenem-resistant *Pseudomonas aeruginosa* bloodstream infections in China: a 9-year trend and multicenter cohort study

**DOI:** 10.3389/fmicb.2023.1137811

**Published:** 2023-05-18

**Authors:** Qing Yuan, Lei Guo, Bin Li, Sheng Zhang, Haiting Feng, Yan Zhang, Meihong Yu, Hangbin Hu, Hongchao Chen, Qing Yang, Tingting Qu

**Affiliations:** ^1^State Key Laboratory for Diagnosis and Treatment of Infectious Diseases, Collaborative Innovation Center for Diagnosis and Treatment of Infectious Diseases, National Clinical Research Center for Infectious Diseases, Zhejiang University School of Medicine First Affiliated Hospital, Hangzhou, Zhejiang, China; ^2^Department of Infection Control, Wenzhou Medical University of the Second Affiliated Hospital, Wenzhou, Zhejiang, China; ^3^Department of Infectious Diseases, The Fourth Affiliated Hospital, Zhejiang University School of Medicine, Yiwu, Zhejiang, China; ^4^Infection Control Department, The First Affiliated Hospital, Zhejiang University School of Medicine, Hangzhou, Zhejiang, China; ^5^Department of Laboratory Medicine, The First Affiliated Hospital, Zhejiang University School of Medicine, Hangzhou, Zhejiang, China

**Keywords:** carbapenem-resistant, *Pseudomonas aeruginosa*, bacteremia, mortality, risk factor, difficult-to-treat resistant

## Abstract

**Objective:**

Bacteremia caused by carbapenem-resistant *Pseudomonas aeruginosa* (CRPA) has high mortality, threatening the healthcare quality worldwide. Analysis is required to update the epidemiological data of CRPA bloodstream infections (BSI) and evaluate the prevalent strains in China. Moreover, it is necessary to clarify the risk factors associated with the development and mortality of CRPA bacteremia.

**Methods:**

This is a 9-year multicenter retrospective study, enrolling 137 patients with CRPA BSI and 137 carbapenem-susceptible *P. aeruginosa* (CSPA) BSI during January 2012 and December 2020. Antimicrobials susceptibility between the two groups were compared. Risk factors of CRPA BSI were identified by binary logistic regression for development and cox regression for mortality. The Kaplan–Meier method was used to compare time to mortality. CRPA and difficult-to-treat resistant *P. aeruginosa* (DTRPA) detection rate was analyzed year-by-year in ZYH.

**Results:**

A total of 7,384 *P. aeruginosa* clinical samples were cultured in ZYH during 9  years, and notable increase of CRPA and DTRPA detection rate in *P. aeruginosa* BSI was identified (from 17 to 60%; from 2.1 to 25%). Multivariate analysis revealed that prior ICU hospitalization, immunosuppressive therapy and exposure to carbapenems were independent risk factors for development of CRPA BSI. The 30-day crude mortality of 137 CRPA BSI was 39%. A total of 46 DTRPA were identified, and the 30-day mortality for patients infected by DTRPA was 50%. The 30-day crude mortality of CRPA BSI was independently associated with multiple organ failure and higher Pitt bacteremia score, whereas receipt appropriate therapy improved prognosis.

**Conclusion:**

A significant increase in the detection rate of CRPA and DTRPA in *P. aeruginosa* BSI was identified. Strict policies for carbapenems usage, cautious decisions regarding the usage of immunosuppressive agent and standard care for patients with prior ICU hospitalization are necessary for CRPA BSI management.

## 1. Introduction

*Pseudomonas aeruginosa* is a major pathogen for healthcare-associated infections, causing different types of infections, such as pneumonia, intra-abdominal infection, urinary tract infection, surgical site infection and bloodstream infections (BSI; Reynolds and Kollef, 2021). Both primary and secondary BSI can lead to severe outcomes and significant socioeconomic burden (Yang et al., 2021). BSI due to *P. aeruginosa* is associated with high morbidity and mortality of approximately 18–45% (Joo et al., 2011; Hirsch et al., 2012; Horino et al., 2012; Patel et al., 2016; Papadimitriou-Olivgeris et al., 2017; Tang et al., 2017; Savaryn et al., 2020; Bergas et al., 2022).

Antimicrobial resistance and the associated delay in appropriate therapy increase the mortality of *P. aeruginosa* BSI (Thaden et al., 2017). Novel antibiotics have been developed for treatment of resistant *P. aeruginosa* in recent years, such as ceftolozane-tazobactam (TOL-TAZ), ceftazidime-avibactam (CAZ-AVI), imipenem-relebactam and cefiderocol (Reynolds and Kollef, 2021). However, carbapenems and typical β-lactam/β-lactamase inhibitor combinations (BLBLIs) are still the predominant agents in clinical practice due to delay marketing approval, high expenses and serious drug side effects of new drugs. Concerningly, carbapenem-resistant *P. aeruginosa* (CRPA) has already become a significant threat to public health worldwide (Tenover et al., 2022). Several molecular mechanisms can result in resistance to carbapenems in Gram-negative bacteria, and the mechanisms vary significantly among different species. Carbapenem resistance in *P. aeruginosa* is predominantly mediated by loss or reduction of porin OprD, overexpression of the cephalosporinase AmpC and efflux pumps (Horcajada et al., 2019). For epidemiological use and practical approach, difficult-to-treat resistant (DTR) has been widely used in recent years. DTR signifies no active first-line drug and even higher level of resistance. Difficult-to-treat resistant *P. aeruginosa* (DTRPA) was defined as non-susceptibility to all of the following antibiotics: ceftazidime, cefepime, ciprofloxacin, levofloxacin, meropenem, imipenem-cilastatin, aztreonam and piperacillin-tazobactam (Kadri et al., 2018).

High morbidity caused by CRPA has been an important clinical concern over the years. The 2020 report of the China Antimicrobial Surveillance Network (CHINET) showed that the isolation rate of CRPA ranged from 23–32% over the past decade (based on imipenem resistance; Hu F. P et al., 2021). European Centre for Disease Prevention and Control (ECDC) and the WHO published 2020 antimicrobial resistance surveillance, 18% carbapenem resistance rate in 20,675 *P. aeruginosa* isolates was reported (Europe, 2022). Previous analysis indicated that carbapenem resistance may increase the mortality of patients infected with *P. aeruginosa* (Liu et al., 2015). Recognizing risk factors for CRPA BSI is important, because it assists physicians to recognize infection and administer proper treatment at an early stage.

In order to clarify the epidemiological trend and antibiotics resistance profile, identify risk factors and prognosis of CRPA BSI in Chinese hospitals. Here, we performed a 9-year multicenter cohort study and provided evidence-based recommendations for infection control and mortality reduction of CRPA BSI in China.

## 2. Methods

### 2.1. Patient and study setting

Our study was conducted in three tertiary hospitals, including the First Affiliated Hospital of Zhejiang University School of Medicine (ZYH, equipped with 2,500 beds), the Fourth Affiliated Hospital of Zhejiang University School of Medicine (ZSH, equipped with 1,100 beds) and the Second Affiliated Hospital of Wenzhou Medical University (WYH, equipped with 2,900 beds) from January 2012 to December 2020. The inclusion criteria consisted of (i) CRPA was detected at least once in blood or intravenous catheter culture; (ii) the first episode of bloodstream culture; (iii) patients could be diagnosed as *P. aeruginosa* BSI (Horan et al., 2008). The primary outcomes were the 30 day mortality. A total of 177 patients with CRPA BSI were identified during the 9 year study period, then 40 cases were excluded due to polymicrobial bacteremia (*n* = 35), incomplete medical record (*n* = 2), or donor-derived infection (*n* = 3). The remaining 137 CRPA BSI patients were eligible for the cohort. 111 patients were from ZYH, 10 from ZSH, and 26 from WYH. Carbapenem-sensitive *P. aeruginosa* (CSPA) BSI cases detected in the current year were randomly matched with CRPA BSI cases, with 1:1 ratio. If the number of CSPA cases was insufficient in the current year, cases that did not enter the cohort study in the adjacent year were randomly selected. Each hospital followed this matching principle. 137 CSPA bacteremia patients were paired. The study flow chart was shown in Supplementary Figure S1.

### 2.2. Study design and data collection

We conducted a retrospective, multicenter cohort study to identify clinical characteristics and risk factors for CRPA BSI. Therapy responses and risk factors associated with CRPA BSI mortality were also evaluated. Epidemiology trends of *P. aeruginosa* isolates in ZYH were analyzed. The data of 274 patients were collected from the electronic medical records. The following data were recorded: antimicrobial susceptibility, demographic, ward, underlying disease, type of resistance, underlying conditions, prior invasive procedure and/or devices, source of bacteremia, exposure to antibiotics within 90 days, conditions after BSI, laboratory examinations, therapeutic medication and mortality.

### 2.3. Definitions

BSI was defined as patients with at least once positive peripheral and/or central line blood culture of *P. aeruginosa,* accompanied by signs and symptoms of infection. CRPA was defined as resistance to imipenem or meropenem (the minimum inhibitory concentration (MIC) ≥ 8 μg/mL), and CSPA was defined as susceptibility to imipenem or meropenem (the MIC≤2 μg/mL; CLSI, 2020). Multidrug-resistant *P. aeruginosa* (MDRPA) was defined as non-susceptibility to at least one agent in three or more antipseudomonal antimicrobials. DTRPA was defined as non-susceptibility to all of the following antimicrobials: ceftazidime, cefepime, ciprofloxacin, levofloxacin, meropenem, imipenem-cilastatin, aztreonam and piperacillin-tazobactam (Tamma et al., 2022). Neutropenia was defined as an absolute neutrophil count <0.5*10^9 cells/L. Appropriate initial antibacterial therapy was defined as receiving one or more antimicrobial agents with *in vitro* activity within 48 h. Appropriate therapy was defined as receiving at least one antimicrobial with *in vitro* activity (Gutiérrez-Gutiérrez et al., 2017). Previous antibiotic therapy was defined as antibiotic used for at least 24 h within the last 90 days before onset of BSI. Glucocorticoid therapy was defined as methylprednisolone consumption at least 40 mg per day (or its equivalent) for more than 48 h. Clinical culture samples of *P. aeruginosa* include: intravenous catheters, bloodstream, feces, urine, bile, cerebrospinal fluid, secretions, drainage fluid, throat swabs, sputum, lavage fluid, brushes, transplant organ preservation fluid, peritoneal fluid and vegetations culture samples.

### 2.4. Microbiology

In this study, microorganisms were identified using the VITEK 2 system (bioMérieux, France). MIC of 12 antipseudomonal agents were performed by the agar dilution method, including ceftazidime, cefepime, ciprofloxacin, levofloxacin, amikacin, gentamicin, tobramycin, imipenem, meropenem, piperacillin-tazobactam, cefoperazone-sulbactam and aztreonam. MIC of polymyxin were performed by the broth microdilution method. The results were interpreted by Clinical and Laboratory Standards Institute standards (CLSI, 2020). Isolates that showed intermediate susceptibility were considered resistant (exclude polymyxin).

### 2.5. Statistical analysis

Chi-square test or Fisher’s exact test was used for categorical variables, and t-test or Mann–Whitney *U*-test for continuous variables to compare groups in our study. Selected variables with *p* values ≤0.20 in univariate analysis were included in multivariate analyses. We used binary logistic regression to evaluate predictors associated with the development of CRPA BSI and used cox regression to identify risk factors associated with the outcomes of CRPA BSI. The Kaplan–Meier method was used to compare time to mortality. All statistical analyses were performed in IBM SPSS Statistics v.23.0. *p* values <0.05 were considered statistically significant.

## 3. Results

### 3.1. Local epidemiological trends and strain characteristics of *Pseudomonas aeruginosa* over a 9 year period

**Figure 1:**
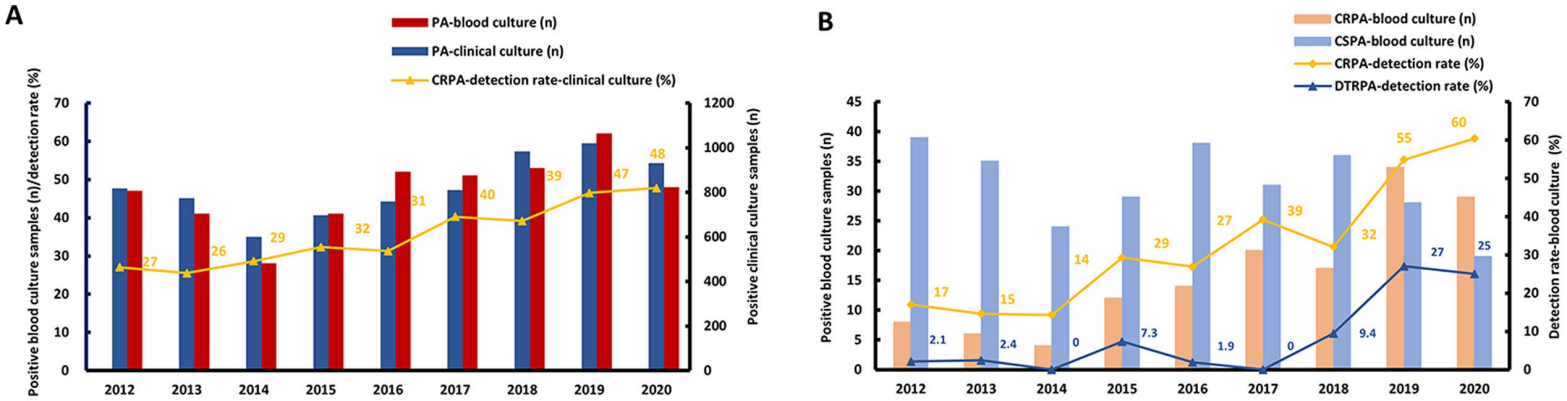
Epidemiological data of *Pseudomonas aeruginosa* (PA) at the first affiliated hospital of Zhejiang university school of medicine: **(A)** Main axis (left): number of positive samples for *P. aeruginosa* blood culture (n) /year, detection rate of CRPA in *P. aeruginosa* clinical culture samples (%)/year; sub-axis (right): number of positive samples for *P. aeruginosa* clinical culture (n)/year. **(B)** Main axis (left): number of positive samples for CRPA and CSPA blood culture (*n*) /year; sub-axis (right): detection rate of CRPA and DTRPA in *P. aeruginosa* blood culture samples (%)/year.

Since ZYH detected the largest number of CRPA BSI cases among the three hospitals, it was chosen for analyzing *P. aeruginosa* epidemiological characteristics. The non-duplicate *P. aeruginosa* and CRPA clinical isolates were collected between January 2012 and December 2020 (Figure 1A). A total of 7,384 *P. aeruginosa* clinical samples were cultured in ZYH during 9 years. From 2014, we recognized a remarkable increase in both *P. aeruginosa* clinical culture and bloodstream culture samples, and the first downward trend was shown by 2020. The proportion of carbapenem resistant isolates among all *P. aeruginosa* clinical culture samples increased notably from 27% in 2012 to 48% in 2020, and annual incidence increased year on year. Since 2012, we found CRPA detection rate in bloodstream culture samples increased dramatically from 17% in 2012, to 55% in 2019, even 60% in 2020 (Figure 1B). Moreover, DTRPA detection rate in bloodstream culture samples increase dramatically from 2.1% in 2012 to 25% in 2020.

### 3.2. Antimicrobial susceptibility of the *Pseudomonas aeruginosa* isolates

Antimicrobial susceptibility of the 274 *P. aeruginosa* isolates is listed in Table 1. Apart from carbapenem resistant phenotypes, the 137 CRPA bloodstream culture isolates in our study also exhibited resistance to ceftazidime, cefepime, ciprofloxacin, levofloxacin, piperacillin-tazobactam, cefoperazone-sulbactam and aztreonam, and the antimicrobial resistance were 60, 56, 51, 54, 45, 62 and 57%, respectively. However, the antimicrobial resistance to amikacin, tobramycin and polymyxin were only 4.1, 8.3 and 5.8%, respectively. Except for amikacin and polymyxin, the remain 11 antibiotics in CRPA group showed significantly higher resistance than that in CSPA isolates. Moreover, the proportion of MDRPA and DTRPA isolates were notably higher in CRPA group than that in CSPA group (68% vs. 7.3%, *p* < 0.001; 34% vs. 0, *p* < 0.001). Forty-six DTRPA isolates were detected among all *P. aeruginosa* isolates (Table 2).

**Table 1:** Antimicrobial susceptibility of the 274 *Pseudomonas aeruginosa* isolates to 13 types of antibiotics (CRPA vs. CSPA).

Antibiotic(s)	CRPA (*n* = 137)		CSPA (*n* = 137)		*p* Value (R%)
	MIC range (μg/mL)	Resistance rate (%)	MIC range (μg/mL)	Resistance rate (%)	
Ceftazidime	2–64	60	1–64	12	**<0.001**
Cefepime	1–64	56	1–64	10	**<0.001**
Ciprofloxacin	0.25–4	51	0.25–2	3.7	**<0.001**
Levofloxacin	0.25–8	54	0.25–4	6.7	**<0.001**
Amikacin	2–64	4.1	2–16	0	0.072
Gentamicin	1–16	18	1–8	1.1	**<0.001**
Tobramycin	1–16	8.3	1–4	0	**0.003**
Imipenem	4–16	100	1–4	5.9	**<0.001**
Meropenem	1–16	87	0.25–4	1.3	**<0.001**
Piperacillin/tazobactam	4–128	45	4–128	13	**<0.001**
Cefoperazone/sulbactam	8–64	62	8–64	18	**<0.001**
Aztreonam	4–64	57	2–64	31	**0.008**
Polymyxin	0.5–8	5.8	0.5–8	1.9	0.536

CRPA, Carbapenem-resistant *Pseudomonas aeruginosa*; CSPA, Carbapenem-sensitive *Pseudomonas aeruginosa*.

Isolates that showed intermediate susceptibility were considered resistance (exclude polymyxin).

**Table 2:** Characteristics of patients with *P. aeruginosa* bloodstream infection, stratified by carbapenems susceptibility.

Characteristic	Total (274)	CRPA (*n* = 137)	CSPA (*n* = 137)	*p* Value
Demographic				
Sex–Male	188 (69%)	96 (70%)	92 (67%)	0.603
Age, years, median (IQR)	62.5 (50, 73)	61 (46.5, 73)	64 (51.5, 73)	0.251
Elderly (age ≥ 55y)	182 (66%)	84 (61%)	98 (72%)	0.073
Prior hospitalization (2 weeks)	140 (51%)	76 (56%)	64 (47%)	0.147
Prior ICU hospitalization^a^	52 (19%)	39 (29%)	13 (9.5%)	**<0.001**
Ward				
ICU	97 (35%)	58 (42%)	39 (29%)	**0.016**
Hematology	48 (18%)	24 (18%)	24 (18%)	1.000
Underlying disease	248 (91%)	126 (92%)	122 (89%)	0.410
Diabetes	53 (19%)	28 (20%)	25 (18%)	0.646
Chronic lung disease	41 (15%)	19 (14%)	22 (16%)	0.611
Chronic renal disease	41 (15%)	22 (16%)	19 (14%)	0.611
Chronic liver disease	45 (16%)	25 (18%)	20 (15%)	0.415
Solid malignant tumor	68 (25%)	25 (18%)	43 (31%)	**0.012**
Cardiovascular diseases	111 (41%)	52 (38%)	59 (43%)	0.389
Cerebrovascular diseases	33 (12%)	15 (11%)	18 (13%)	0.578
Solid-organ transplant	12 (4.4%)	8 (5.8%)	4 (2.9%)	0.238
Hematological disease	67 (25%)	33 (24%)	34 (25%)	0.888
Benign biliary diseases	45 (16%)	21 (15%)	24 (18%)	0.625
Trauma	16 (5.8%)	9 (6.6%)	7 (5.1%)	0.606
Type of resistance				
MDRPA	103 (38%)	93 (68%)	10 (7.3%)	**<0.001**
DTRPA	46 (17%)	46 (34%)	0	**<0.001**
Underlying medical conditions				
Length hospital stay before infection, days, median (IQR)	11 (3, 25)	16 (6, 29)	8 (2, 17)	**<0.001**
ERCP/PTCD Surgery^b^	31 (11%)	17 (12%)	14 (10%)	0.567
Glucocorticoid therapy	91 (33%)	56 (41%)	35 (26%)	**0.007**
Immunosuppressive therapy	59 (22%)	40 (29%)	19 (14%)	**0.002**
Prior invasive procedure and/or devices^a^	212 (77%)	116 (85%)	96 (70%)	**0.004**
Mechanical ventilation	111 (41%)	66 (48%)	45 (33%)	**0.010**
Urinary catheterization	132 (48%)	68 (50%)	64 (47%)	0.629
CVC	158 (58%)	93 (68%)	65 (47%)	**0.001**
Percutaneous catheterization	70 (26%)	39 (29%)	31 (23%)	0.268
Source of bacteremia				
Lung	61 (22%)	32 (23%)	29 (21%)	0.663
Skin and soft-tissue	10 (3.6%)	6 (4.4%)	4 (2.9%)	0.519
Biliary tract	44 (16%)	19 (14%)	25 (18%)	0.324
Urinary tract	22 (8.0%)	12 (8.8%)	10 (7.3%)	0.657
Catheter related	27 (9.9%)	15 (11%)	12 (8.8%)	0.543
Intra-abdominal	7 (2.6%)	2 (1.5%)	5 (3.6%)	0.444
Surgical sites	8 (2.9%)	4 (2.9%)	4 (2.9%)	1.000
Unknown	94 (34%)	46 (34%)	48 (35%)	0.799
Exposure to anti-infectives within 90 days				
Tigecycline	41 (15%)	29 (21%)	12 (8.8%)	**0.004**
Polymyxin	9 (3.3%)	8 (5.8%)	1 (0.7%)	**0.042**
BLBLIS	122 (45%)	73 (53%)	49 (36%)	**0.004**
Ceftazidime-avibactam	4 (1.5%)	4 (2.9%)	0	0.131
Carbapenems	132 (48%)	93 (68%)	39 (29%)	**<0.001**
Meropenem	62 (23%)	53 (39%)	9 (6.6%)	**<0.001**
*Daily dose, grams*	3.00 (1.50, 3.00)	3.00 (1.50, 3.00)	3.00 (2.13, 3.00)	0.484
*Total dose, grams*	21.00 (6.00, 33.00)	24.00 (6.00, 34.50)	12.75 (5.25, 21.00)	0.180
Imipenem	58 (21%)	34 (25%)	24 (18%)	0.139
Biapenem	30 (11%)	20 (15%)	10 (7.3%)	0.053
Faropenem	4 (1.5%)	1 (0.7%)	3 (2.2%)	0.614
Cephalosporins	90 (33%)	40 (29%)	50 (37%)	0.198
Aminoglycosides	90 (33%)	40 (29%)	50 (37%)	0.198
Quinolones	11 (4.0%)	7 (5.1%)	4 (2.9%)	0.356
The condition after BSI				
MOF	67 (25%)	44 (32%)	23 (17%)	**0.003**
Sepsis or septic shock	94 (34%)	59 (43%)	35 (26%)	**0.002**
Mechanical ventilation	91 (33%)	55 (40%)	36 (26%)	**0.015**
Total length of hospital stay, days, median (IQR)	27 (13.75, 45)	31 (17.5, 52.5)	21 (11, 34.5)	**<0.001**
Medical expenses, RMB	100155.40 (44112.39, 227431.17)	147573.35 (69653.92, 313945.00)	68900.88 (28757.67, 153281.00)	**<0.001**
Laboratory examinations				
Neutrophilic granulocyte, median (IQR)^c*^	8.45 (1.33, 13.08)	8.78 (1.25, 14.10)	8.26 (1.30, 12.60)	0.532
Hemoglobin, median (IQR)^d⁜^	83 (67, 105)	79 (66, 97)	87 (70, 107)	**0.028**
Platelet, median (IQR)^d*^	118.5 (31.25, 202.5)	110 (21.5, 226)	123 (42, 198)	0.746
C-reactive protein, median (IQR)^e▴^	113.60 (61.09, 167.21)	113.00 (64.19, 174.91)	114.13 (53.30, 166.44)	0.395
Procalcitonin, median (IQR)^e⁕^	2.43 (0.49, 17.59)	2.66 (0.48, 15.35)	2.08 (0.52, 30.49)	0.846
Agranulocytosis	60 (22%)	33 (24%)	27 (20%)	0.381
PBS, median (IQR)	2 (1, 5)	2 (1, 6)	1 (1, 3)	**0.018**
Mortality				
All-cause death at 7 d	60 (22%)	37 (27%)	23 (17%)	**0.041**
All-cause death at 14 d	74 (27%)	43 (31%)	31 (23%)	0.103
All-cause death at 30 d	94 (34%)	54 (39%)	40 (29%)	0.075

Data are presented as no. (%) unless otherwise indicated. Bolded numbers indicate that *p* < 0.05.

CRPA, Carbapenem-Resistant *Pseudomonas aeruginosa*; CSPA, Carbapenem-sensitive *P. aeruginosa*; IQR, interquartile range; ICU, intensive care unit; RMB, Renminbi; ERCP, Endoscopic Retrograde Cholangio-Pancreatography; PTCD, Percutaneous transhepatic cholangial drainage; CVC, central venous catheter; MDRPA, multidrug-resistant *P. aeruginosa*; DTRPA, difficult-to-treat resistant *P. aeruginosa*; BLBLIS, β-lactam/β-lactamase Inhibitor Combinations, including Piperacillin-tazobactam and Cefoperazone-sulbactam; BSI, bloodstream infection; MOF, Multiple organ failure; PBS, Pitt bacteremia score.

Units: ^*^ 0.5 × 10^9 cells/L; ^⁜^ g/L; ^▴^ mg/L, ^⁕^ ng/mL.

a. Evaluated 30 days before the first positive blood culture.

b. During hospitalization or 2 weeks before admission.

c. Evaluated 48 h before and after the first positive blood culture, whichever is the highest or lowest when out of the normal range.

d. Evaluated 48 h before and after the first positive blood culture, whichever is the lowest.

e. Evaluated 48 h before and after the first positive blood culture, whichever is the highest.

### 3.3. Demographic and clinical characteristics of patients with *Pseudomonas aeruginosa* bloodstream infection in multicenter analysis

The baseline characteristics of 274 *P. aeruginosa* BSI patients were presented in Table 2. Most patients were male (69%, *n* = 188) and elderly older than 55 years (66%, *n* = 182). 51% (*n* = 140) of the patients had history of admission at least once prior to their first positive culture. The majority of patients came from ICU (35%, *n* = 97) and the hematology ward (18%, *n* = 48). Cardiovascular disease (mainly hypertension) presented 41% (*n* = 111) of the underlying disease, followed by solid malignant tumor (25%, *n* = 68), hematological diseases (25%, *n* = 67), diabetes (19%, n = 53). Among patients with hematological diseases, 53(79%) were acute leukemia or lymphoma. Underlying disease were not statistically different between CRPA and CSPA group, implying that the baseline general conditions were balanced. Except for unknown origin of infection, lung (22%), biliary tract source (16%), catheter related infection (9.9%) and urinary tract source (8.0%) were the most frequent infection sites, and this trend was consistent across the two groups.

### 3.4. Risk factors of development of CRPA bacteremia

In the univariate analysis of patients with CRPA and CSPA BSI, the results revealed that patients infected by CRPA tended to had prior ICU hospitalization, longer hospital stay before infection, glucocorticoid therapy, immunosuppressive therapy, receipt of mechanical ventilation and central venous catheter (CVC), lower hemoglobin, higher Pitt bacteremia score (PBS), and poor prognosis after BSI (including multiple organ failure (MOF), *p* = 0.003; sepsis/septic shock, *p* = 0.002; underwent mechanical ventilation, *p* = 0.015; longer total length of hospital stay, *p* < 0.001; higher medical expenses, *p* < 0.001).

Moreover, hospital stay before infection in CRPA group was one-fold than CSPA group [16 (6, 29) days vs. 8 (2, 17) days; *p* < 0.001]. The difference of antibiotics exposed within 90 days prior to recovery between carbapenem resistance group and carbapenem susceptibility group lay mainly in carbapenems (68% vs. 29%, *p* < 0.001), meropenem (39% vs. 6.6%, *p* < 0.001), BLBLIs(53% vs. 36%, *p* = 0.004)and tigecycline (21% vs. 8.8%, *p* = 0.004). Neither daily dose [3.00 (1.50, 3.00) grams vs. 3.00 (2.13, 3.00) grams; *p* = 0.484) nor total consumption [24.00 (6.00, 34.50) grams vs. 12.75 (5.25, 21.00 grams; *p* = 0.180)] of meropenem expose was different between the two groups.

When entering the multivariate logistic analysis, prior ICU hospitalization (OR 4.160; 95% CI 1.985–8.719; *p* < 0.001), immunosuppressive therapy (OR 2.112; 95% CI 1.061–4.203; *p* = 0.033), and exposure to carbapenems within 90 days (including meropenem, imipenem, biapenem) were independent risk factors associated with development of CRPA bacteremia (Table 3).

**Table 3:** Risk factors for development, 7-day and 30-day mortality in patients with carbapenem-resistant *P. aeruginosa* bloodstream infection based on multivariate analysis.

Variable	OR/HR (95% CI)	*p* Value
CRPA BSI		
Prior ICU hospitalization	4.160 (1.985, 8.719)	**<0.001**
Immunosuppressive therapy	2.112 (1.061, 4.203)	**0.033**
Exposure to meropenem within 90 days	8.821 (3.990, 19.501)	**<0.001**
Exposure to imipenem within 90 days	2.103 (1.090, 4.056)	**0.027**
Exposure to biapenem within 90 days	2.512 (1.026, 6.151)	**0.044**
7-day mortality		
Hematological diseases	2.785 (1.349, 5.750)	**0.006**
PBS	1.193 (1.092, 1.304)	**<0.001**
MOF	3.825 (1.493, 9.798)	**0.005**
Appropriate therapy	0.327 (0.154, 0.693)	**0.004**
30-day mortality		
PBS	1.092 (1.009, 1.182)	**0.030**
MOF	7.098 (3.435, 14.667)	**<0.001**
Appropriate therapy	0.312 (0.164, 0.593)	**<0.001**

CRPA, Carbapenem-Resistant *Pseudomonas aeruginosa*; ICU, intensive care unit; PBS, Pitt bacteremia score; MOF, Multiple organ failure.

### 3.5. Outcomes of *Pseudomonas aeruginosa* bloodstream infection

**Figure 2:**
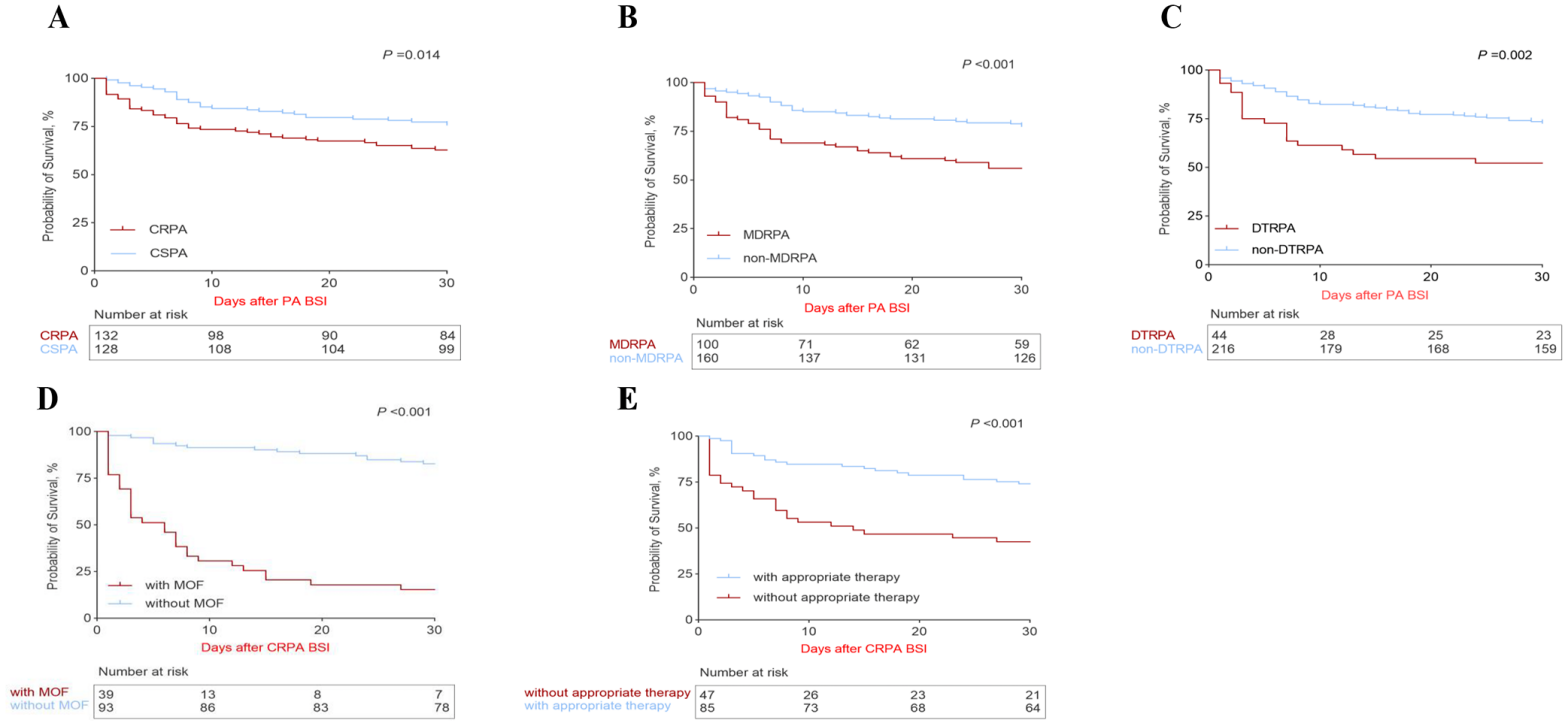
Kaplan–Meier curves of the estimated 30-day probability of survival for 274 *P. aeruginosa* bloodstream infection (PA BSI) patients caused by carbapenem-resistant *P. aeruginosa* (CRPA) and carbapenem-susceptible *P. aeruginosa* (CSPA) **(A)**; with multidrug-resistant (MDR) type and non-MDR type **(B)**; with difficult-to-treat resistant (DTR) type and non-DTR type **(C)**. Kaplan–Meier curves of the estimated 30-day probability of survival for 137 CRPA BSI patients with and without multiple organ failure (MOF) **(D)**; with and without appropriate therapy **(E)**.

During the 30 days follow-up, 94 (34%) of 274 *P. aeruginosa* infected patients died and 23 (50%) of 46 DTRPA infected patients died. The 7 day, 14 day and 30 day crude mortality in CRPA group and CSPA group were 27% vs. 17% (*p* = 0.041), 31% vs. 23% (*p* = 0.103), 39% vs. 29% (*p* = 0.075), respectively. Distinction between the two groups became inapparent as the disease progressed for more than 2 weeks. Of 274 eligible patients in our study, 201 (73%) received appropriate therapy after BSI onset, whereas that rate of CRPA BSI patients died within 7 day and 30 day were only 35 and 41%, respectively. The median PBS of CRPA BSI patients died within 7 days was 6 and that patients deceased within 30 days was 5; the median PBS was 2 of surviving patients. In 274 *P. aeruginosa* BSI patients, the 30-day survival probability of patients with CRPA BSI was significantly worse than that of those with CSPA BSI [Hazard Ratio (HR) 1.732, 95% CI 1.117–2.686, *p* = 0.014, log-rank test; Figure 2A]. The survival of patients with MDRPA BSI was significantly worse than that of patients with non-MDRPA BSI (HR 2.301, 95% CI 1.452–3.646, *p* < 0.001; Figure 2B). The survival of patients with DTRPA BSI was significantly worse than that of patients with non-DTRPA BSI (HR 2.138, 95% CI 1.143–4.000, *p* = 0.002; Figure 2C).

### 3.6. Risk factors for mortality of CRPA bloodstream infection

The univariate analysis for 7-day crude mortality of CRPA BSI was shown in Supplementary Table S1. Multivariate cox regression analysis (Table 3) identified the following as independent risk factors for 7-d mortality: hematological disease (HR 2.785; 95% CI 1.349–5.750, *p* = 0.006), higher PBS (HR 1.193; 95% CI 1.092–1.304, *p* < 0.001), MOF (HR 3.825; 95% CI 1.493–9.798, *p* = 0.005). Receipt of appropriate therapy was identified as independent factor associated with better outcome (HR 0.327; 95% CI 0.154–0.693, *p* = 0.004).

The univariate analysis revealed that the patients deceased within 30 days tend to had hematological disease, glucocorticoid therapy, MOF and sepsis or septic shock, lower platelet, higher C-reactive protein and procalcitonin, agranulocytosis and higher PBS (Table 4). No association between different sources of CRPA bacteremia and 30-day mortality. In multivariate cox regression (Table 3), the 30-day crude mortality of CRPA BSI was independently associated with MOF (HR 7.098; 95% CI 3.435–14.667; *p* < 0.001) and higher PBS (HR 1.092; 95% CI 1.009–1.182; *p* = 0.030), whereas receipt appropriate therapy improved prognosis (HR 0.312; 95% CI 0.164–0.593; *p* < 0.001). This conclusion was consistent with the results of the survival analysis (Figures 2D,E).

**Table 4:** Characteristics of patients with carbapenem-resistant *P. aeruginosa* bloodstream infection, stratified by 30-day outcome.

Characteristic	Total (137)	Non-survivors (*n* = 54)	Survivors (*n* = 83)	*p* Value
Demographic				
Sex–Male	96 (70%)	35 (65%)	61 (74%)	0.278
Age, years, median (IQR)	61 (46.5, 73)	57 (43.75, 69.75)	62 (49, 75)	0.151
Elderly (age ≥ 55y)	84 (61%)	27 (50%)	57 (69%)	**0.028**
Underlying disease	126 (92%)	49 (91%)	77 (93%)	0.916
Diabetes	29 (21%)	6 (11%)	23 (28%)	**0.020**
Chronic lung disease	19 (14%)	8 (15%)	11 (13%)	0.796
Chronic renal disease	22 (16%)	5 (9.3%)	17 (21%)	0.080
Chronic liver disease	25 (18%)	7 (13%)	18 (22%)	0.196
Solid malignant tumor	25 (18%)	7 (13%)	18 (22%)	0.196
Cardiovascular diseases	52 (38%)	23 (43%)	29 (35%)	0.367
Cerebrovascular diseases	15 (11%)	6 (11%)	9 (11%)	0.961
Solid-organ transplant	8 (5.8%)	2 (3.7%)	6 (7.2%)	0.626
Hematological disease	33 (24%)	21 (39%)	12 (15%)	**0.001**
Benign biliary diseases	21 (15%)	11 (20%)	10 (12%)	0.186
Trauma	9 (6.6%)	2 (3.7%)	7 (8.4%)	0.460
Type of resistance				
MDRPA	93 (68%)	41 (76%)	52 (63%)	0.104
DTRPA	46 (34%)	23 (43%)	23 (28%)	0.071
Underlying medical conditions				
ERCP/PTCD Surgery^a^	17 (12%)	4 (7.4%)	13 (16%)	0.152
Glucocorticoid therapy	56 (41%)	31 (57%)	25 (30%)	**0.001**
Immunosuppressive therapy	40 (29%)	20 (37%)	20 (24%)	0.104
Source of bacteremia				
Lung	32 (23%)	12 (22%)	20 (24%)	0.800
Skin and soft-tissue	6 (4.4%)	2 (3.7%)	4 (4.8%)	1.000
Biliary tract	19 (14%)	5 (9.3%)	14 (17%)	0.208
Urinary tract	12 (8.8%)	3 (5.6%)	9 (11%)	0.447
Catheter related	15 (11%)	5 (9.3%)	10 (12%)	0.609
Intra-abdominal	2 (1.5%)	2 (3.7%)	0	0.154
Surgical sites	4 (2.9%)	1 (1.9%)	3 (3.6%)	0.937
Unknown	46 (34%)	23 (43%)	23 (28%)	0.071
The condition after BSI				
MOF	44 (32%)	38 (70%)	6 (7.2%)	**<0.001**
Sepsis or septic shock	59 (43%)	39 (72%)	20 (24%)	**<0.001**
Mechanical ventilation	55 (40%)	25 (46%)	30 (36%)	0.236
Laboratory examination				
Platelet, median (IQR)^b*^	110 (21.5, 226)	23 (9, 130.25)	159 (65, 250)	**<0.001**
C-reactive protein, median (IQR)^c▴^	113.0 (64.2, 174.9)	132.8 (82.3, 200.0)	105.5 (62, 148.5)	**0.049**
Procalcitonin, median (IQR)^c⁕^	2.66 (0.48, 15.35)	12.16 (1.89, 27.60)	1.36 (0.45, 11.64)	**0.008**
Agranulocytosis	33 (24%)	21 (39%)	12 (15%)	**0.001**
PBS, median (IQR)	2 (1, 6)	5 (1, 11.25)	2 (0, 4)	**<0.001**
Antibiotics usage after infection				
Appropriate initial therapy within 48 h	54 (39%)	17 (32%)	37 (45%)	0.125
Appropriate therapy	85 (62%)	22 (41%)	63 (76%)	**<0.001**
Combined definitive therapy	56 (41%)	21 (39%)	35 (42%)	0.703
Carbapenem+ Quinolones	4 (2.9%)	3 (5.6%)	1 (1.2%)	0.338
Carbapenem+ polymyxin	2 (1.5%)	2 (3.7%)	0	0.154
BLBLIS+ Quinolones	10 (7.3%)	2 (3.7%)	8 (9.6%)	0.333
BLBLIS+ polymyxin	4 (2.9%)	1 (1.9%)	3 (3.6%)	0.937
BLBLIS+ Aminoglycosides	5 (3.6%)	1 (1.9%)	4 (4.8%)	0.661

Data are presented as no. (%) unless otherwise indicated. Bolded numbers indicate that *p* < 0.05.

IQR, interquartile range; ICU, intensive care unit; ERCP, Endoscopic Retrograde Cholangio-Pancreatography; PTCD, Percutaneous transhepatic cholangial drainage; MDRPA, multidrug-resistant *Pseudomonas aeruginosa*; DTRPA, difficult-to-treat resistant *Pseudomonas aeruginosa*; BLBLIS, β-lactam/β-lactamase Inhibitor Combinations, including Piperacillin-tazobactam and Cefoperazone-sulbactam; BSI, bloodstream infection; MOF, Multiple organ failure; PBS, Pitt bacteremia score.

Units: ^*^ 0.5 × 10^9 cells/L; ^▴^ mg/L, ^⁕^ ng/mL.

a. During hospitalization or 2 weeks before admission.

b. Evaluated 48 h before and after the first positive blood culture, whichever is the lowest.

c. Evaluated 48 h before and after the first positive blood culture, whichever is the highest.

## 4. Discussion

In 2016, a priority list of antibiotic-resistant bacteria was created by WHO for research support and development of effective drugs. Not surprisingly, CRPA was listed as one of the critical-priority tiers (Tacconelli et al., 2018). Since bacterial pathogens are constantly evolving and adapting, it is essential to update the epidemiological data of *P. aeruginosa* and evaluate locally prevalent strains. Our study recognized remarkable changes in drug resistance phenotype and increase in CRPA and DTRPA prevalence from bloodstream culture samples during 2012–2020. Thus, active bloodstream culture surveillance of CRPA is necessary for guiding effective antibacterial therapy in clinical practice (Tacconelli et al., 2014). In a recently published article, we studied the characteristics of CRPA bloodstream isolates in ZYH from 2019 to 2020 (Hu H. et al., 2021). The results showed that 48% isolates belonging to a novel high-risk *Klebsiella pneumoniae* carbapenemase (KPC)-producing Sequence Type (ST) 463, and all these strains can be classified as DTRPA type. Moreover, all ST463 isolates coharbored *exoU* and *exoS* genes, which are the most clinically relevant effectors in the type III secretion system for increasing the virulence and resistance in *P. aeruginosa* infections (Horna et al., 2019; Reynolds and Kollef, 2021). The molecular mechanism provides an explanation for notably increase in CRPA and DTRPA detection rate from bloodstream culture isolates in our study.

We demonstrated that carbapenem-resistant, multidrug-resistant and difficult-to-treat resistant can increase mortality in *P. aeruginosa* BSI patients. Carbapenem-resistant strains are often resistant to other antimicrobials such as β-lactams and quinolones (Codjoe and Donkor, 2017), consistent with drug susceptibility results in our study. Inappropriate antibiotic choice has previously been proven to be associated with increased mortality among patients with *P. aeruginosa* bloodstream infection (Micek et al., 2005). Our study confirms that receipt appropriate therapy improved 7-day and 30-day outcomes among patients with CRPA BSI. According to antimicrobial susceptibility results, polymyxin and aminoglycosides are recommended drugs. Since CAZ-AVI entered the market in China in May 2019, *in vitro* susceptibility analysis of CAZ-AVI was rarely done in our study. But based on background that China is a prevalent country with KPC producing *P. aeruginosa* epidemic, the application of CAZ-AVI has played a promising role (Kazmierczak et al., 2016; Hu Y. et al., 2021). TOL-TAZ is a novel BLBLIs, which was first approved by the FDA in 2014 and not yet available in China (as of April 2023). TOL-TAZ has high activity against *P. aeruginosa* by enhancing affinity for the penicillin binding proteins and significantly less affected by the changes in the porin permeability or efflux pumps (Cho et al., 2015). Study have shown a sensitivity of 85% for CRPA, and also recommended TOL-TAZ for the treatment of CRPA BSI (Farrell et al., 2013). Since KPC cannot be inhibited by tazobactam (van Duin and Bonomo, 2016), the sensitivity of CRPA isolates to TOL-TAZ is uncertain in our study.

Identifying antimicrobial susceptibility profile and risk factors of CRPA BSI could be vital for providing prompt appropriate therapy. Some studies have identified the potential predictors for CRPA BSI. Consistent with published case–control (Tuon et al., 2012; Valderrama et al., 2016; Li et al., 2018) and cohort (Kang et al., 2005; Barron et al., 2016; Lee et al., 2017; Shi et al., 2019; Kang et al., 2021) studies, we identified that previous exposure to carbapenems (including meropenem, imipenem and biapenem) was an independent risk factor, but the dose of drug exposure is not related to.

Besides previous carbapenems use, ICU stay before BSI onset and immunosuppressive therapy are associated with the development of CRPA. Patients had prior ICU hospitalized experience are at particularly high risk of severe disease, invasive medical procedures and long-term consumption of antibiotics. *P. aeruginosa* is also a critical important pathogen in immunocompromised patients, particularly patients with hematological malignancies (Cattaneo et al., 2012). In this study, the majority of immunosuppressive agents were treated for hematological malignancies. Comparing independent risk factors of 7-day and 30-day mortality in patients with CRPA BSI, we demonstrated that hematological disease could increase the short-term mortality. High-dose combined chemotherapy with resultant skin and mucosa damage can provide a convenient port of entry for pathogens during nadir stage (Zhao et al., 2020). Bone marrow suppression and outer barrier damage caused by chemotherapy that progresses to agranulocytosis also increases the risk of sepsis and lead to severe outcome. Therefore, strict policies for carbapenems usage, cautious decisions regarding the usage of immunosuppressive agent and standard care for patients with prior ICU hospitalization are necessary for CRPA BSI management.

The PBS has been used to measure acute severity of illness and predict mortality in patients with BSI. Patients have been classified as critically ill with PBS ≥4 due to a higher mortality risk (Al-Hasan and Baddour, 2020). Our finding was consistent with this classification, and we demonstrated that higher PBS was independent risk factors for 7-day and 30-day mortality. Previous studies demonstrated that the initial site of surgery infections and pneumonia were associated with worse outcome for *P. aeruginosa* infections (Reynolds and Kollef, 2021). However, no association between different sources of CRPA bacteremia and 30-day mortality in our study.

This study had some limitations due to the properties of retrospective and observational research. First, patients were collected from a single province, and two hospitals (ZSH and WYH) had very small cases due to low *P. aeruginosa* BSI prevalence. However, the three hospitals are located in the north of Zhejiang (ZYH), central Zhejiang (ZSH), and southern Zhejiang (WYH), which are representatives in Zhejiang Province. Moreover, ZYH often attracts patients from surrounding provinces to seek treatment, with hematology and infectious diseases as specialties. Thus, our findings still have better application to other regions in eastern China. Second, due to delay marketing approval in China, the number of cases in which CAZ-AVI has been applied is limited. Although we demonstrated the association between previous carbapenems therapy and CRPA BSI, antibiotic used before admission to the hospital might not be accurate. Therefore, further large-scale and well-designed prospective multicenter study are warranted.

Our study showed that the detection rate of CRPA and DTRPA bloodstream culture samples has increased dramatically in recent years. Prior ICU hospitalization, immunosuppressive therapy and exposure to carbapenems were independent risk factors for development of CRPA BSI. Appropriate therapy can significantly reduce mortality rate, but hematological diseases increase the 7-day mortality.

## Data availability statement

The original contributions presented in the study are included in the article/Supplementary material, further inquiries can be directed to the corresponding author.

## Author contributions

TQ was responsible for the initial draft and analysis. QYu, LG, and BL: collection and collation the clinical data. SZ, HF, and HH: epidemiological data acquisition and statistical analysis. QYu, YZ, and MY: manuscript writing and graphics drawing. TQ, QYa, and HC: manuscript revisions and approved the final manuscript. All authors contributed to the article and approved the submitted version.

## Conflict of interest

The authors declare that the research was conducted in the absence of any commercial or financial relationships that could be construed as a potential conflict of interest.

## Publisher’s note

All claims expressed in this article are solely those of the authors and do not necessarily represent those of their affiliated organizations, or those of the publisher, the editors and the reviewers. Any product that may be evaluated in this article, or claim that may be made by its manufacturer, is not guaranteed or endorsed by the publisher.
