# Supplementary material for: Risk factors and outcomes of inpatients with carbapenem-resistant Pseudomonas aeruginosa bloodstream infections in China: a 9-year trend and multicenter cohort study
Source: Front Microbiol. 2023 May 18;14:1137811. doi: 10.3389/fmicb.2023.1137811 (PMC10227572; doi:10.3389/fmicb.2023.1137811)
Supplement: Supplementary file 2 [file Table_2.DOCX]

**Supplementary Table 1.** **Characteristics of** **patients with carbapenem-resistant *P. aeruginosa* bloodstream infection, stratified by 7-day outcome**

| **Characteristic** | **Total (137)** | | | **Non-survivors (n=37)** | **Survivors (n=100)** | ***P* Value** |
| --- | --- | --- | --- | --- | --- | --- |
| **Demographic** |  | | |  |  |  |
| Sex–Male | 96 (70%) | | | 28 (76%) | 68 (68%) | 0.384 |
| Age, years, median (IQR) | 61 (46.5, 73) | | | 53 (40, 68.5) | 64 (48, 75) | **0.032** |
| Elderly (age≥55y) | 84 (61%) | | | 16 (43%) | 68 (68%) | **0.008** |
| **Underlying disease** | 126 (92%) | | | 35 (95%) | 91 (91%) | 0.739 |
| Diabetes | 28 (20%) | | | 2 (5.4%) | 26 (26%) | **0.008** |
| Chronic lung disease | 19 (14%) | | | 4 (11%) | 15 (15%) | 0.529 |
| Chronic renal disease | 22 (16%) | | | 3 (8.1%) | 19 (19%) | 0.123 |
| Chronic liver disease | 25 (18%) | | | 4 (11%) | 21 (21%) | 0.170 |
| Solid malignant tumor | 25 (18%) | | | 2 (5.4%) | 23 (23%) | **0.018** |
| Cardiovascular diseases | 52 (38%) | | | 16 (43%) | 36 (36%) | 0.438 |
| Cerebrovascular diseases | 15 (11%) | | | 4 (11%) | 11 (11%) | 1.000 |
| Solid-organ transplant | 8 (5.8%) | | | 1 (2.7%) | 7 (7.0%) | 0.588 |
| Hematological disease | 33 (24%) | | | 18 (49%) | 15 (15%) | **<0.001** |
| Benign biliary diseases | 21 (15%) | | | 5 (14%) | 16 (16%) | 0.720 |
| Trauma | 9 (6.6%) | | | 2 (5.4%) | 7 (7.0%) | 1.000 |
| **Type of resistance** |  | | |  |  |  |
| MDRPA | 93 (68%) | | | 30 (81%) | 63 (63%) | **0.044** |
| DTRPA | 46 (34%) | | | 18 (49%) | 28 (28%) | **0.023** |
| **Underlying medical conditions** | |  | |  |  |  |
| ERCP/PTCD Surgery^a^ | 17 (12%) | | | 1 (2.7%) | 16 (16%) | 0.071 |
| Glucocorticoid therapy | 56 (41%) | | | 23 (62%) | 33 (33%) | **0.002** |
| Immunosuppressive therapy | 40 (29%) | | | 16 (43%) | 24 (24%) | **0.028** |
| **Source of bacteremia** |  | | |  |  |  |
| Lung | 32 (23%) | | | 8 (22%) | 24 (24%) | 0.770 |
| Skin and soft-tissue | 6 (4.4%) | | | 1 (2.7%) | 5 (5.0%) | 0.910 |
| Biliary tract | 19 (14%) | | | 1 (2.7%) | 18 (18%) | **0.021** |
| Urinary tract | 12 (8.8%) | | | 3 (8.1%) | 9 (9.0%) | 1.000 |
| Catheter related | 15 (11%) | | | 3 (8.1%) | 12 (12%) | 0.734 |
| Intra-abdominal | 2 (1.5%) | | | 0 | 2 (2.0%) | 1.000 |
| Surgical sites | 4 (2.9%) | | | 1 (2.7%) | 3 (3.0%) | 1.000 |
| Unknown | 46 (34%) | | | 19 (51%) | 27 (27%) | **0.007** |
| **The condition after BSI** |  | | |  |  |  |
| MOF | 44 (32%) | | | 29 (78%) | 15 (15%) | **<0.001** |
| Sepsis or septic shock | 59 (43%) | | | 27 (73%) | 32 (32%) | **<0.001** |
| Mechanical ventilation | 55 (40%) | | | 19 (51%) | 36 (36%) | 0.104 |
| **Laboratory examination** |  | | |  |  |  |
| Neutrophilic granulocyte, median (IQR)^b*^ | 8.78 (1.25, 14.10) | | | 1.00 (0, 12.10) | 9.95 (5.03, 14.58) | **0.002** |
| Hemoglobin, (mean, SD)^c⁜^ | 82.76 (23.15) | | | 75.19 (20.06) | 85.56 (23.67) | **0.019** |
| Platelet, median (IQR)^c*^ | 110 (21.5, 226) | | | 15 (9, 46.5) | 150 (58, 247) | **<0.001** |
| C-reactive protein, median▴ (IQR)^d^ | 112.96 (64.19, 174.91) | | | 154.89 (82.30, 212.30) | 105.50 (62.00, 151.38) | **0.043** |
| Procalcitonin, median (IQR)^d⁕^ | 2.66 (0.48, 15.35) | | | 4.57 (1.08, 29.39) | 1.62 (0.46, 13.58) | 0.112 |
| Agranulocytosis | 33 (24%) | | | 18 (49%) | 15 (15%) | **<0.001** |
| PBS, median (IQR) | 2 (1, 6) | | | 6 (2, 12) | 2 (0, 4) | **<0.001** |
| **Antibiotics usage after infection** | | |  |  |  |  |
| Appropriate initial therapy with 48h | 54 (39%) | | | 9 (24%) | 45 (45%) | **0.028** |
| Appropriate therapy | 85 (62%) | | | 13 (35%) | 72 (72%) | **<0.001** |
| Combined definitive therapy |  | | |  |  |  |
| Carbapenem+Quinolones | 4 (2.9%) | | | 2 (5.4%) | 2 (2.0%) | 0.631 |
| Carbapenem+ polymyxin | 1 (0.7%) | | | 1 (2.7%) | 0 | 0.270 |
| BIBLIs+Quinolones | 10 (7.3%) | | | 0 | 10 (10%) | 0.104 |
| BIBLIs+polymyxin | 4 (2.9%) | | | 0 | 4 (4.0%) | 0.507 |
| BIBLIs+Aminoglycosides | 5 (3.6%) | | | 1 (2.7%) | 4 (4.0%) | 1.000 |

Data are presented as no. (%) unless otherwise indicated. Bolded numbers indicate that *P* < 0.05.

Abbreviations: IQR, interquartile range; ICU, intensive care unit; ERCP, Endoscopic Retrograde Cholangio-Pancreatography; PTCD, Percutaneous transhepatic cholangial drainage; MDRPA, multidrug-resistant *Pseudomonas aeruginosa*; DTRPA, difficult-to-treat resistant *Pseudomonas aeruginosa*; BLBLIS, β-lactam/β-lactamase Inhibitor Combinations, including Piperacillin-tazobactam and Cefoperazone-sulbactam; BSI, bloodstream infection; MOF, Multiple organ failure; PBS, Pitt bacteremia score.

Units: ^*^ 0.5 × 10^9 cells/L; ^⁜^ g/L; ^▴^ mg/L, ^⁕^ ng/ml.

1. During hospitalization or 2 weeks before admission.
2. Evaluated 48 hours before and after the first positive blood culture, whichever is the highest or lowest when out of the normal range.
3. Evaluated 48 hours before and after the first positive blood culture, whichever is the lowest.
4. Evaluated 48 hours before and after the first positive blood culture, whichever is the highest.
