# Supplementary figures and images for: Risk factors and outcomes of inpatients with carbapenem-resistant Pseudomonas aeruginosa bloodstream infections in China: a 9-year trend and multicenter cohort study
Source: Front Microbiol. 2023 May 18;14:1137811. doi: 10.3389/fmicb.2023.1137811 (PMC10227572; doi:10.3389/fmicb.2023.1137811)

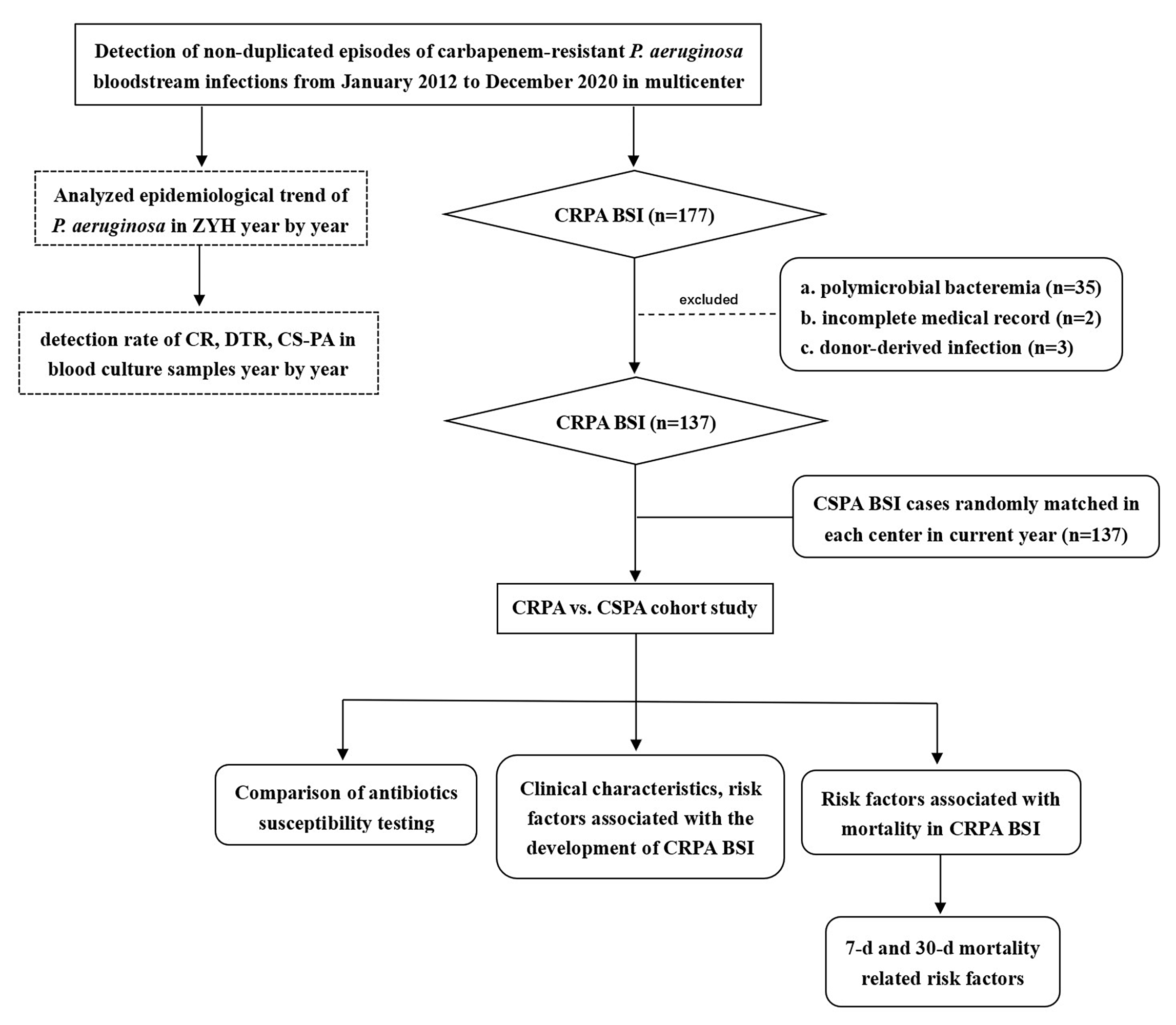

Supplement: Supplementary file 3 [file Image_1.TIF]
